# Impact of Antibiotic-Loaded PMMA Spacers on the Osteogenic Potential of hMSCs

**DOI:** 10.3390/antibiotics13010044

**Published:** 2024-01-03

**Authors:** Jakob Hofmann, Tim Niklas Bewersdorf, Ulrike Sommer, Thomas Lingner, Sebastian Findeisen, Christian Schamberger, Gerhard Schmidmaier, Tobias Großner

**Affiliations:** 1 Clinic for Trauma and Reconstructive Surgery, Centre for Orthopedics, Trauma and Reconstructive Surgery and Paraplegiology, University Hospital Heidelberg, Schlierbacher Landstrasse 200a, 69118 Heidelberg, Germany; jakob.hofmann@med.uni-heidelberg.de (J.H.); timniklas.bewersdorf@med.uni-heidelberg.de (T.N.B.); sebastian.findeisen@med.uni-heidelberg.de (S.F.);; 2 Genevention GmbH, Rudolf-Wissell-Str. 28A, 37079 Goettingen, Germany

**Keywords:** osteogenesis, anti-infective agents, PMMA bone cement, ^99m^Tc-HDP labelling, gentamicin, vancomycin, clindamycin, non-union, bone defect

## Abstract

Antibiotic-loaded PMMA bone cement is frequently used in modern trauma and orthopedic surgery. Although many of the antibiotics routinely applied are described to have cytotoxic effects in the literature, clinical experience shows no adverse effects for bone healing. To determine the effects of antibiotic-loaded PMMA spacers on osteogenesis in vitro, we cultivated human bone marrow mesenchymal stem cells (BM-hMSCs) in the presence of PMMA spacers containing Gentamicin, Vancomycin, Gentamicin + Clindamycin as well as Gentamicin + Vancomycin in addition to a blank control (agarose) and PMMA containing no antibiotics. The cell number was assessed with DAPI staining, and the osteogenic potential was evaluated by directly measuring the amount of hydroxyapatite synthesized using radioactive ^99m^Tc-HDP labelling as well as measuring the concentration of calcium and phosphate in the cell culture medium supernatant. The results showed that Gentamicin and Vancomycin as well as their combination show a certain amount of cytotoxicity but no negative effect on osteogenic potential. The combination of Gentamicin and Clindamycin, on the other hand, led to a drastic reduction in both the cell count and the osteogenic potential.

## 1. Introduction

The treatment of bone infections and related bone defects is a frequent, yet complex, challenge in modern orthopaedic and trauma surgery [1]. Particularly, infection-related non-unions are difficult to treat, often requiring multiple revision surgeries over the course of weeks or even months [2]. Bone infections show a relatively high incidence, especially after trauma (infections after fracture fixation (IAFF)), where they occur in up to 30% of open grade III° tibia fractures [3,4,5]. Infections can also arise from elective bone-related surgery. Prosthetic joint infections (PJIs), a possible complication of total hip arthroplasty as well as total knee arthroplasty, occur in up to 2.5% of these treatments [6,7].

Whether an infection can be attributed to IAFF or PJI, the best option for treatment consists of a radical debridement of the bone and the removal of all permanent foreign material (plates, nails, prothesis), always in combination with a high-dose antibiotic treatment [2,8]. For the latter, antibiotics can either be administered intravenously or locally via elution from drug-loaded spacers or antibiotic beats [8,9,10]. When administered locally, higher concentrations of the anti-infective agent can be reached without increasing the risk of systemic side effects associated with i.v. administration [11,12]. This is especially important for the treatment of bone infections attributed to *Staphylococci,* which are capable of forming biofilms as their effective treatment requires much higher concentrations of anti-infective agents [13].

Alternatively, anti-infective agents can be released from a vast number of materials, including bio-gels and polymers such as polymethyl-methacrylate (PMMA), which is the material most often used for bone cement today, e.g., for prophylactic reasons for the fixation of prosthetic joint implants, as spacers to create a biological membrane for the two-step treatment of segmental bone defects (Masquelet technique), or for the consistent local delivery of high doses of antibiotics in the treatment of infected bones and infected non-unions [14,15,16,17]. Bone cement is usually provided as a two-phase material, where one phase (usually a powder) contains a PMMA prepolymer and a polymerization initiator and the other phase (usually a liquid) contains MMA monomers, an accelerator and a stabiliser [18,19]. By mixing these two phases, the exothermic polymerization is initiated, finally leading to the formation of PMMA bone cement [20].

Two of the most commonly used anti-infective agents in drug-loaded spacers are Gentamicin and Clindamycin as they are effective against a wide range of bacteria frequently found in bone infections, including *Staphylococci* [17,21,22]. As methicillin-resistant strands of *Staphylococcus aureus* (MRSA) have become more prevalent during recent years, the glycopeptide Vancomycin has also become increasingly important, being the typical treatment for MRSA infections [23,24,25].

Several antibiotics, including Gentamycin and Clindamycin, have been shown to be cytotoxic, especially in high concentrations [26]. This would lead to an impaired osteogenesis, prolonging the healing process. However, in vitro data suggest that, whereas Gentamicin is especially effective in reducing the cell count of a cell culture when added directly to the cell culture medium, there is no reduction in overall osteogenic potential due to a relatively higher osteogenic activity in the cells [27]. 

The question remaining is whether these effects only exist for the direct application of anti-infective agents solved in the cell culture medium or if they also occur when anti-infective agents are eluted from PMMA spacers, as happens in clinical applications in the human body.

The osteogenic potential of cell cultures is usually defined by their ability to form hydroxyapatite [28,29]. To assess the amount of hydroxyapatite, the highly sensitive, novel method of ^99m^Tc-HDP labelling allows for a rapid quantitative evaluation of the mineral deposition. Here, a radioactive tracer (^99m^Tc) bound to a polyphosphate (HDP) directly binds to newly formed hydroxyapatite while this uptake can be visualized with a gamma camera and precisely quantified using an activimeter [30]. Beyond that, this is a non-destructive way to measure the amount of hydroxyapatite formed so the cell cultures remain intact for further assessment [31].

The purpose of this study is to assess whether anti-infective agents eluted from PMMA bone cement have any negative effect on the osteogenic potential of human mesenchymal stem cells in vitro. The osteogenic potential is, hereby, represented by the cell number as well as the cell’s ability to form hydroxyapatite.

## 2. Results

### 2.1. DAPI Cell Count

**Figure 1:**
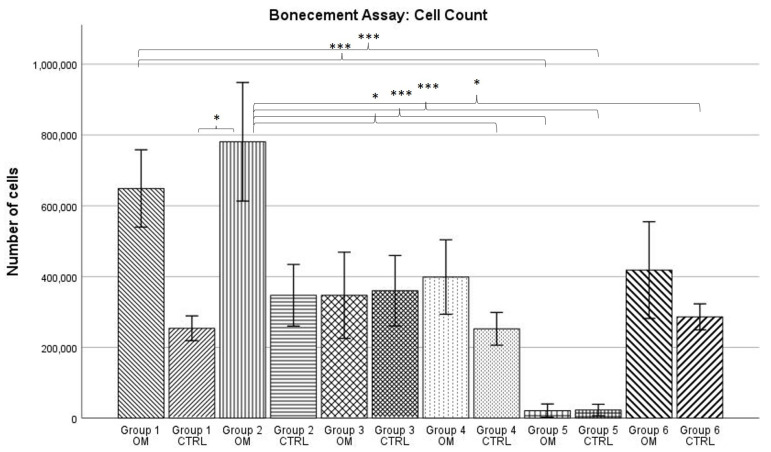
Mean cell count per group as established by DAPI cell count. The error bars show the error of mean. Bars connected by brackets are significantly different from one another. Stars indicate the level of significance: *** = *p* < 0.001; * = *p* ≤ 0.05. “OM” stands for osteogenic differentiation, “CTRL” for negative control.

For both the osteogenic and the control conditions, the number of cells for most groups averaged between 200,000 and 400,000 per dish (see Figure 1). However, there were two notable exceptions. The highest mean number of cells was counted in osteogenic group 2 (PMMA with no antibiotics) with 780,814 cells, closely followed by osteogenic group 1 (no PMMA, no antibiotics) with 648,925 cells. Still, although these numbers were higher than the cell count of the osteogenic conditions of all other cell counts, no significance could be found, with the exception of group 5 (Gentamicin + Clindamycin). In the osteogenic condition of this group, the lowest cell count of all groups could be counted, with a mean of 21,252 cells per dish. This was almost paralleled by the mean of 23,020 cells per dish for the corresponding control condition. Subsequently, the ANOVA analysis revealed a statistically significant difference between group 5 (osteogenic and control condition) and the osteogenic condition of groups 1 and 2 (*p* < 0.001).

Further significant differences could be shown between group 2, osteogenic condition and the control conditions of groups 1, 4, 5, and 6 as well as group 1, osteogenic condition and the control conditions of groups 4 and 5. For all groups, the data showed normal distribution according to the Kolmogorov–Smirnov test.

### 2.2. ^99m^Tc-HDP Labelling

**Figure 2:**
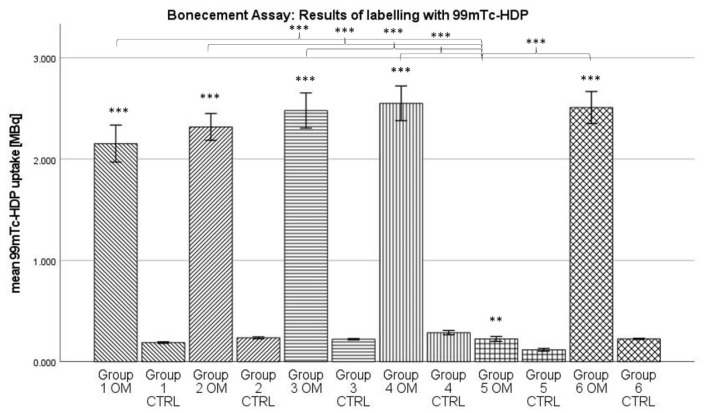
Mean ^99m^Tc-HDP uptake. The error bars show the error of mean. Columns connected by brackets are significantly different from one another. Stars over Columns indicate the statistical difference between the osteogenic und control conditions of one group. Stars indicate the level of significance: *** = *p* < 0.001; ** = *p* < 0.01. “OM” stands for osteogenic differentiation, “CTRL” for negative control.

For the osteogenic condition, the highest ^99m^Tc-HDP mean uptake could be measured for group 4, with 2.551 MBq, while the lowest was shown by group 5, with 0.225 MBq (see Figure 2). The same trend was observed for the control condition, where the highest ^99m^Tc-HDP uptake was shown in group 4 with 0.287 MBq and the lowest in group 5 with 0.118 MBq.

For all groups, the data showed normal distribution according to the Kolmogorov–Smirnov test. The ANOVA analysis revealed the following: Firstly, when looking at the osteogenic conditions, groups 1–4 and group 6 showed a significantly higher uptake than group 5 (*p* < 0.001). Secondly, when looking at the control conditions, all groups showed a statistically similar uptake. For every group, the osteogenic condition showed a significantly higher uptake than the control condition (groups 1–4 and 6: *p* < 0.001; group 5: *p* < 0.01).

### 2.3. Calcium in Cell Culture Medium

**Figure 3:**
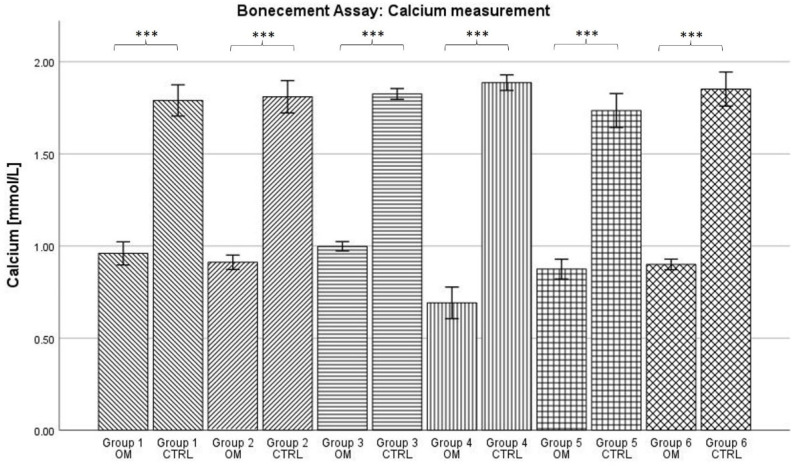
Mean concentration of calcium in cell culture medium per group. The error bars show the error of mean. Bars connected by brackets are significantly different from one another. Stars indicate the level of significance: *** = *p* < 0.001. “OM” stands for osteogenic differentiation, “CTRL” for negative control.

Calcium concentration in the cell culture medium was always higher in the control condition for every group (see Figure 3). For the osteogenic conditions, calcium concentration ranged from 0.69 mmol/L for group 4 to 1.00 mmol/L for group 3. For the control conditions, calcium concentrations were between 1.74 mmol/L for group 5 and 1.89 mmol/L for group 4.

For all groups, the data showed normal distribution according to the Kolmogorov–Smirnov test. The ANOVA analysis showed that the calcium concentration was statistically significantly higher in the control condition compared to the osteogenic condition for each group (*p* < 0.001). However, no further significances were detected.

### 2.4. Phosphate in Cell Culture Medium

**Figure 4:**
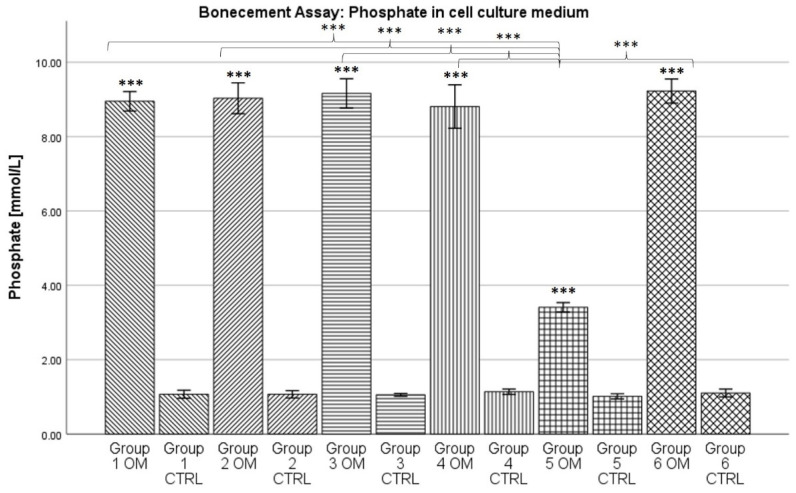
Mean concentration of phosphate in cell culture medium per group. The error bars show the error of mean. Columns connected by brackets are significantly different from one another. Stars over Columns indicate the statistical difference between the osteogenic und control conditions of one group. Stars indicate the level of significance: *** = *p* < 0.001. “OM” stands for osteogenic differentiation, “CTRL” for negative control.

In opposition to the results for calcium concentration in the cell culture medium, the phosphate concentration was always higher in the osteogenic condition (see Figure 4). Here, it ranged from 3.41 mmol/L for group 5 to 9.23 mmol/L for group 6. The phosphate concentration for the control conditions ranged from 1.01 mmol/L for group 5 to 1.14 mmol/L for group 4.

For all groups, the data showed normal distribution according to the Kolmogorov–Smirnov test. The ANOVA analysis revealed a statistically significant difference between the osteogenic condition and its corresponding control condition for every group (*p* < 0.001). Furthermore, the phosphate concentration in the medium of the osteogenic condition of group 5 was significantly lower than the concentration for the osteogenic conditions of all other groups (*p* < 0.001).

## 3. Discussion

Our data showed that drug-loaded PMMA spacers do have an influence on the osteogenic potential and the cell number of BM-hMSC cultures, although this influence is strongly dependent on the antibiotic used. Moreover, the effects on the osteogenic potential and effects on the cell count vary from one another.

The DAPI cell count showed that under the influence of most anti-infective agents, the cell count is between 200,000 and 400,000 cells per dish. Groups 1 and 2, in which no antibiotic was used, showed cell numbers about twice as high, between 600,000 and 800,000 cells per dish. This trend is, however, not statistically significant, although that might be at least partially mathematical in nature as cell counts often show a high variance. Still, when comparing the cell counts of groups containing antibiotics (groups 3–6) and groups without antibiotics (groups 1 and 2), it seems clear that a reduction in the cell count takes place. This same effect could be shown when directly adding antibiotics to the cell culture medium: the presence of Gentamicin and Vancomycin led to a reduction in the number of cells in the culture [27]. Therefore, two things can be concluded: Firstly, Vancomycin and Gentamicin are cytotoxic, especially at higher doses. This is a known effect and well described in the literature [32,33]. Secondly, the mode of administration (added to cell culture media or eluted out of PMMA cement) does not seem to influence the general effect of cytotoxicity. A reduction in cell number could be observed for both direct addition of antibiotics to the cell culture medium as well as indirect addition via elution from drug-loaded PMMA bone cement.

A much more dramatic reduction in the cell number than for Vancomycin and Gentamicin was seen under the influence of a drug-eluting PMMA spacer containing Clindamycin and Gentamicin (group 5). Here, the reduction in the cell number was not only statistically significant from groups 1 and 2 (*p* < 0.001); in some samples, not a single cell could be identified in the DAPI cell count. The cytotoxic effect of Clindamycin in vitro appears to be much more pronounced than the one of Gentamicin and Clindamycin. When looking at the literature, one can find a number of studies confirming the cytotoxic effects of Clindamycin [34,35]. A study by Naal et al. determined that when looking specifically at osteoblasts, proliferation rates could be reduced to 3.5% of what could be measured in the negative control [35]. While the dose at which cytotoxic effects start to take place is not higher for Clindamycin than for Vancomycin or Gentamicin, Clindamycin’s chemical structure might help to explain the much more pronounced reduction in cell number [32,33,34]. Clindamycin is a very lipophilic molecule and can, therefore, much more easily penetrate the membranes of osteoblasts and their predecessor stem cells when compared to the more hydrophilic Gentamicin and Vancomycin [36]. As a consequence, Clindamycin concentrations are higher in bone tissue when compared to the other two antibiotics [37]. Furthermore, under certain conditions, the effectiveness of Clindamycin can be increased. This is mainly used as a wanted effect, e.g., for the eradication of *Propionibacterium acnes* to treat acne [38]. However, an increase in Clindamycin effectiveness might also lead to an increased cytotoxicity, e.g., towards stem cells. One of the substances able to increase Clindamycin’s effectiveness is Benzoyl peroxide (BPO) [39]. BPO can also serve as a polymerization initiator and, as such, is a component of Palacos bone cement (1%) [40,41]. Therefore, Clindamycin eluted from PMMA spacers might be more cytotoxic than Clindamycin alone. In addition, BPO itself shows antibiotic properties as it is able to generate free radicals, making PMMA bone cement possibly cytotoxic [38,42]. These concerns were strong enough that, recently, BPO-free bone cements have been developed [40].

Another observation that can be made when looking at the data from the DAPI cell count is the higher number of cells for the osteogenic conditions compared to the control conditions for groups 1 and 2. Although, again, not statistically significant, it could be explained by the presence of dexamethasone in the osteogenic medium. Dexamethasone is known to promote the proliferation of MSCs in doses within the nanomole/L range, which is the concentration we used for differentiation (see Section 4.6) [39]. However, why the presence of antibiotics stops the pro-proliferatory effects of dexamethasone so that there is no difference in cell number between osteogenic and control conditions for groups 3 to 6 has yet to be explained.

Overall antibiotic-eluting PMMA spacers have a relatively large negative influence on the cell count of the BM-hMSC culture in vitro. When looking at the osteogenic potential, determined by ^99m^Tc-HDP labelling, this negative influence is much less pronounced.

The ^99m^Tc-HDP labelling showed a very similar uptake for most groups regarding their osteogenic conditions. When only looking at the results of the ^99m^Tc-HDP labelling, it is impossible to see the underlying difference in cell number. This, however, has one exception: for group 5 (Gentamicin + Clindamycin), the radiotracer uptake is significantly lower compared to all other groups. For Gentamicin and Vancomycin, it seems that the reduction in cell number does not lead to a reduction in osteogenic potential. These results are consistent with earlier findings [27]. In a study from 2021, we showed that osteogenically differentiated BM-hMSCs cultures always showed an uptake of around 3.5 MBq, whether antibiotics were added to the cell culture medium or not. While it is tempting to explain the lower uptake value in our current study with the cytotoxic effects of PMMA bone cement [42], one has to keep in mind that those two studies may not be compared directly, as the culture conditions were not identical (for example, different cell donors were used). More important than the absolute uptake value is, instead, the relation between groups within one study, and here we see that in both cases, the ^99m^Tc-HDP uptake is largely independent from the presence of Vancomycin and Gentamycin. Therefore, the osteogenic potential seems far more stable towards the effects of certain antibiotics than the cell number. Whether this is due to an increase in the metabolic rate of the individual cell or due to other effects is yet to be determined. Whatever the effects responsible, for Clindamycin, they do not seem potent enough to compensate for the steep reduction in cell number, leading to a significantly lower osteogenic potential.

During ^99m^Tc-HDP labelling, all groups showed a significantly higher uptake of radiotracer for the osteogenic condition when compared to the control group. This can be seen as proof of a successful osteogenesis [29,30], even when the various antibiotics were present. These results are supported by the assessment of the concentrations of calcium and phosphate in the cell culture medium. The reduction in calcium concentration is known to be an inverse non-invasive marker for osteogenesis [43]. In our data, the calcium concentration was significantly lower for all osteogenic conditions when compared to the control conditions. Vice versa, high phosphate concentrations probably correlate with osteogenesis [27]. In our data, the phosphate concentration was always significantly higher for the osteogenic groups. In summary, it can be stated that the formation of calcium complexes took place in the osteogenically differentiated groups. As earlier studies showed, these calcium complexes are equivalent in structure to hydroxyapatite [31]. Therefore, a successful osteogenesis took place.

In summary, most antibiotics frequently used in clinical practice appear to show a moderate negative effect of the cell number in vitro, reducing it to about 50% compared to cell cultures where non-antibiotics are added. However, this effect was not statistically significant. The ^99m^Tc-HDP uptake, as a marker of hydroxyapatite formation, and, thus, of osteogenic potential, was very similar between most groups, indicating that the antibiotics and their combinations used had no negative effect on the osteogenic potential of MSCs, with the exception of Clindamycin. Clindamycin, on the other hand, shows a profoundly negative on both the cell number and the osteogenic potential. Both decreased dramatically in comparison to all other groups, whether their culture conditions included antibiotics or not.

Our study is limited by the fact that it was conducted in vitro and can, therefore, not be transferred directly into clinical practice. Some mechanisms may differ in the living organism, especially when it comes to enzymatic activity or the anti-inflammatory effects of other cells. For example, the contamination of bone cement with physiological fluids such as blood is known to potentially influence its biomechanical properties [44]. Still, our results may serve as a basis for following in vivo studies, further assessing this highly relevant aspect of septic bone surgery. In the end, these studies may serve to finally prove the biological safety of anti-infective agents eluted by PMMA bone cement, adding an important background of scientific evidence to current clinical experience.

## 4. Materials and Methods

### 4.1. Study Design at a Glance

In this study, we examined how the osteogenic potential of bone marrow mesenchymal stem cells in vitro is influenced by the presence of antibiotic-loaded bone cement spacers frequently used in clinical practice. For this, we used PMMA cement and the antibiotics Gentamicin, Vancomycin and Clindamycin.

Bone marrow aspirate was harvested from the proximal femoral cavity of *n* = 6 healthy donors during elective hip surgery. The mononuclear cell fraction in this aspirate was isolated using a Ficoll gradient. After isolation, plastic-adherent cells were cultivated in T-150 polystyrene tissue culture flasks using DMEM HG until 90% confluence was reached. Expansion continued until a sufficient number of cells was available.

**Figure 5:**
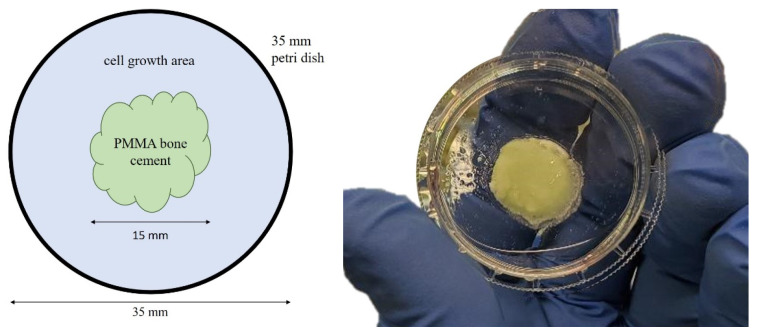
Principle of Petri dish preparation (**left**) and example of a prepared Petri dish (**right**).

Following this, 144 35 mm flat-bottom Petri dishes were prepared by blocking a radius of r = 0.75 cm with one of the following substances (see Figure 5):

- Group 1: agarose (2 × 6 dishes osteogenic differentiation, 2 × 6 dishes control)
- Group 2: 0.5 g PMMA bone cement (2 × 6 dishes osteogenic differentiation, 2 × 6 dishes control)
- Group 3: 0.5 g PMMA bone cement containing Gentamicin (2 × 6 dishes osteogenic differentiation, 2 × 6 dishes control)
- Group 4: 0.5 g PMMA bone cement containing Vancomycin (2 × 6 dishes osteogenic differentiation, 2 × 6 dishes control)
- Group 5: 0.5 g PMMA bone cement containing Gentamicin und Clindamycin (2 × 6 dishes osteogenic differentiation, 2 × 6 dishes control)
- Group 6: 0.5 g PMMA bone cement containing Gentamicin und Vancomycin (2 × 6 dishes osteogenic differentiation, 2 × 6 dishes control)

The dimension of the PMMA circle was determined in a preliminary experiment, which revealed a weight of 0.5 g of bone cement to result in the intended antibiotic concentration. Based on this weight and the Petri dish height, the 0.75 cm radius was calculated. To ensure identical growth conditions, especially regarding total available surface area, the same area was blocked in group 1, using agarose instead of PMMA, which served as a blank.

Around this central circle, cells were seeded with a density of 10,000 cells/cm^2^ and cultivated using DMEM LG. Half of these dishes were osteogenically differentiated using the osteogenic supplements L-ascorbic acid, dexamethasone and β-glycerol phosphate. The other half served as control and did not receive osteogenic supplements in its medium.

Osteogenic differentiation continued for 21 days. After 7, 14 and 21 days, calcium and phosphate concentrations were measured in the medium supernatant. On day 21, the cell culture was terminated. Half of the dishes were used to assess the amount of hydroxyapatite formed by means of the results assessed by ^99m^Tc-HDP labelling. For this, the dishes were incubated with 5 MBq of ^99m^Tc-HDP for 15 min. After washing, the bound activity was measured using an activimeter.

To evaluate the number of cells, the second half of the dishes was DAPI stained. After that, the number of cells was counted under a fluorescence microscope.

### 4.2. Harvest of Human Mesenchymal Stem Cells

Bone marrow aspirate was obtained from *n* = 6 healthy donors from their proximal femoral cavity under general anaesthesia during the elective implantation of a total hip endoprosthesis after informed consent was given. During the preparation of the proximal bone cavity, 10 mL of bone marrow was aspirated into a 20 mL syringe (BD, Heidelberg, Germany) which contained 1000 IU of heparin (Braun, Melsungen, Germany). The samples were subsequently diluted 1:1 with PBS (Gibco, Frankfurt, Germany) and washed twice with PBS. For the isolation of the mononuclear cell fraction, a Ficoll gradient centrifugation (Ficoll-Paque-PLUS, Cytiva, Freiburg, Germany) was used. The mononuclear cells were then seeded into T-150 polystyrene tissue culture flasks (Falcon, Kaiserslautern, Germany) with a density of $5\times 10^{5}$ cells/cm^2^. The cells were cultured in a humidified 5% CO_2_ atmosphere at 37 °C. As medium, Dulbecco’s modified Eagle’s medium (DMEM-HG, Gibco) was used, containing 10% heat-inactivated (56 °C, 30 min) fetal bovine serum (FCS, Sigma, Schnelldorf, Germany) and 1% penicillin/streptomycin (Sigma). After 48 h, the culture flasks were washed with PBS to remove non-adherent cells. The remaining cells were defined as human bone marrow mesenchymal stem cells (hMSCs). Medium was changed three times a week (every 2–3 days) until 90% confluence was reached. The cells were then trypsinised (Trypsin-EDTA, Sigma) and stored as P0 cells until needed in liquid nitrogen. One aliquot for storage contained $5\times 10^{5}$ cells in 0.5 mL DMSO (Sigma).

### 4.3. hMSC Expansion

P0 hMSCs (*n* = 6) were thawed and seeded into T-150 flasks (Falcon) with a density of ${5\times 10}^{5}$ cells/cm^2^ and then cultured in a humidified atmosphere (5% CO_2_, 37 °C). Medium was changed every 2 days. Expansion medium was DMEM-HG with 10% FCS and 1% penicillin/streptomycin. After 90% confluence was reached in a cell cultures, the cells were trypsinised.

### 4.4. Determination of Bone Cement Mass

To determine the ideal bone cement mass, a preliminary experiment was conducted. Here, Petri dishes (*n* = 6) were prepared with different amounts of PMMA bone cement containing Gentamicin (Palacos G, Heraeus, Hanau, Germany). Between 0.1 g and 1 g in 0.1 g increments of bone cement was used. Further, 1 mL of cell culture medium (DMEM-LG, Gibco) was added to each dish and incubated at 5% CO_2_ and 37 °C for 48 h. The medium was then removed, and Gentamicin concentration was analysed by the Central Laboratory of Heidelberg University Hospital using a Siemens Dimension EXL200 (Siemens, Erlangen, Germany). Based on these results, a calibration curve was drawn, which showed a necessary weight of 0.5 g PMMA containing Gentamicin for the goal concentration of 60 µg/mL, a typical concentration for the local application of Gentamicin [45,46]. To ensure identical culture conditions, 0.5 g of PMMA bone cement was used for all groups.

### 4.5. Preparation of Petri Dishes

To enable the generation of standardised circles, segments were cut from a 20 mL tube (Falcon). These were then sterilised and subsequently used as a template. A sterile template was placed in the middle of a 35 mm Petri dish and then filled with one of the following substances (for specification of bone cement composition, see Table 1):

- Group 1: agarose (Sigma-Aldrich, Darmstadt, Germany)
- Group 2: 0.5 g of PMMA bone cement (Palacos R, Heraeus, Hanau, Germany)
- Group 3: 0.5 g of PMMA bone cement containing Gentamicin (Palacos R + G, Heraeus)
- Group 4: 0.5 g of PMMA bone cement (Heraeus) mixed with 12 mg Vancomycin (Sigma)
- Group 5: 0.5 g of PMMA bone cement containing Gentamicin and Clindamycin (Copal G + C, Heraeus)
- Group 6: 0.5 g of PMMA bone cement containing Gentamicin and Vancomycin (Copal G + V, Heraeus)

**Figure 6:**
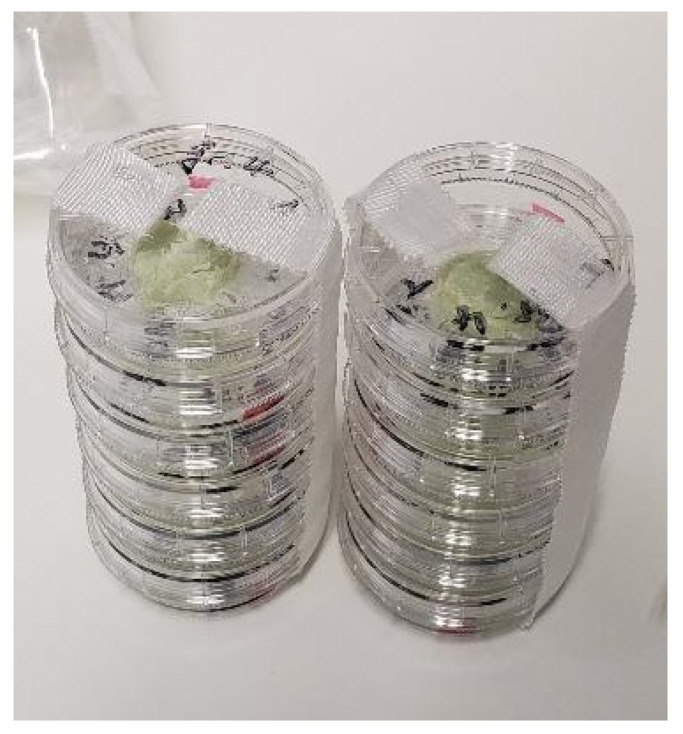
Petri dishes after preparation.

For each group and donor, 4 Petri dishes were prepared (see Figure 6) of which 2 were differentiated osteogenically, while the other two served as negative control.

PMMA bone cements were mixed according to the instructions provided by the producer. Then, 0.5 g of bone cement powder was placed inside the template and mixed with 250 µL of monomer liquid using a sterile spatula until the bone cement reached a homogenous consistency. The template was then carefully removed, and the bone cement was left to harden under a cell culture hood. For group 4, Vancomycin was dosed in such a way that the resulting mass concentration was comparable to the pre-mixed products (1 g of anti-infective agent for 43 g of cement).

### 4.6. hMSC Differentiation

Cells from each donor (*n* = 6) were seeded into 35 mm flat-bottom Petri dishes (Sarstedt, Nümbrecht, Germany) at a density of 10,000 cells/cm^2^ around the central circle (see Section 4.5). In total, 144 dishes were seeded, 24 per group. Half of those dishes were treated with osteogenic medium, which consisted of DMEM-LG containing 10% FCS, 1% penicillin/streptomycin and osteogenic supplements (100 nM dexamethasone (Sigma), 50 µM L-ascorbic acid (Sigma), 10 nM β-glycerol-phosphate (Sigma)) [50]. The other half of dishes served as controls and were only treated with DMEM-LG containing 10% FCS and 1% penicillin/streptomycin.

**Figure 7:**
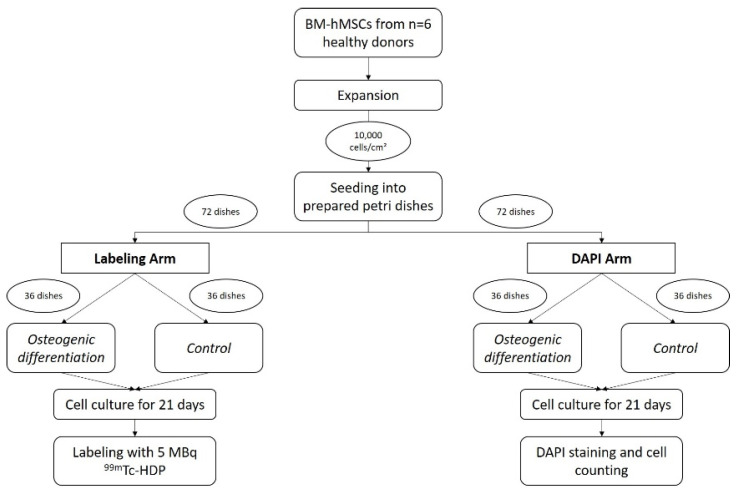
Schematic of the study design.

For differentiation, the culture was running for 21 days in humidified 5% CO_2_ atmosphere at 37 °C. Medium was changed every 2 days. After 21 days, the cell culture was terminated by being washed twice with PBS before air drying under a cell culture hood (for overview of the study design, see Figure 7).

### 4.7. DAPI Staining and Cell Count

Following the termination of the cell culture, dishes assigned to cell counting (*n* = 6, 72 dishes total) were incubated with 4% paraformaldehyde (PFA, Sigma) at room temperature for 20 min. They were then fixated with 0.1% PFA and subsequently stained with DAPI (Thermo Fisher Scientific, Karlsruhe, Germany) before being counted under a microscope (Leica CMi8, Leica, Wetzlar, Germany). Cells were counted using a 100× magnification in 4 vision fields, which corresponded to 4 different areas of the dish (top left, bottom left, top right, bottom right). Based on the specifications of the microscope provided by the producer, the size of one vision field was calculated to be 952.484 µm^2^. Since the area of an entire 35 mm dish was known, the absolute number of cells in the dish could be calculated and assessed for normal distribution.

Cell counting in the fluorescence images was performed using the “CellProfiler” software (Broad Institute, URL: https://cellprofiler.org, version 4.2.4 accessed on 10 October 2022). Raw TIFF images were masked for time stamp and size legend before being converted to greyscale images in which cells were identified as primary objects using a global, manually set intensity threshold (*t =* 0.1). Objects smaller than 8 or bigger than 80 pixels in diameter were disregarded. Clumped cells were distinguished based on the shape method.

### 4.8. ^99m^Tc-HDP Labelling

Following the termination of the cell culture, dishes assigned to ^99m^Tc-HDP labelling (*n* = 6, 72 dishes total) received 5.0 MBq of activity solved in an aliquot of 1 mL of 0.9% NaCl. The dishes were then incubated 15 min at room temperature, before the activity was removed and the dishes were washed twice with PBS. Bound activity was measured with a dose calibrator (Activimeter ISOMED 1010, Nuklearmedizintechnik Dresden, Dresden, Germany). Here, the dishes were placed directly in the detection chamber of the dose calibrator, and the detection window was set to Gamma Decay/^99m^Technetium. Detection time was 5 s for each dish.

### 4.9. Measurements of Calcium and Phosphate in Cell Culture Medium

The supernatant medium collected after 7, 14 and 21 days was thawed and then analysed by the Central Laboratory of Heidelberg University Hospital using a Siemens Dimension EXL200 (Siemens, Erlangen, Germany). Here, the medium concentrations for calcium and phosphate were measured using validated methods for analysing cell culture medium.

### 4.10. Measurements of Calcium and Phosphate in Cell Culture Medium

All results were first tested for normal distribution using the Kolmogorov–Smirnov test. For the determination of statistical significance between the groups, an ANOVA was performed followed by an adjustment of the *p*-value using the post hoc test from Bonferroni. Homogeneity of variances for a one-way ANOVA was analysed using Levene’s test for the assessment of variances.

Statistical analysis was performed using SPSS Statistics^®^ (IBM, Armonk, NY, USA) Version 29. Statistical significance was set to p≤0.05.

## 5. Conclusions

Our data show that, although antibiotics eluted from PMMA may have a cytotoxic effect in vitro, the stem cell’s ability to form bone is not inhibited in most cases, which aligns with clinical experience. In the presence of PMMA eluting Gentamicin and/or Vancomycin, the cells seem to be able to compensate a lower cell number and secrete hydroxyapatite at a rate comparable to antibiotic-free environments. Clindamycin, however, massively reduces the cell count and the osteogenic potential, possibly due to its lipophilic qualities.

## Figures and Tables

**Table 1:** Composition of bone cements used. Percentages are given if provided by manufacturer [41,47,48,49].

Bone Cement Name	Powder Ingredients	Liquid Ingredients
Palacos R	Poly(methylacrylate, methyl methacrylate) (PMMA), zirconium dioxide, benzoyl peroxide (BPO), colorant E141	Methyl methacrylate, N,N-dimethyl-p-toluidine, hydroquinone, colorant E141
Palacos R + G	82% PMMA copolymer, 15% zirconium dioxide, 1% BPO, 2% Gentamicin sulfate, colorant E141	98% Methyl methacrylate, 2% N,N-dimethyl-p-toluidine, hydroquinone, colorant E141
Copal G + C	PMMA copolymer, zirconium dioxide, BPO, 2% Gentamicin sulfate, 2% Clindamycin hydrochloride, colorant E141	Methyl methacrylate, N,N-dimethyl-p-toluidine, hydroquinone, colorant E141
Copal G + V	78% PMMA copolymer, 14% zirconium dioxide, 1% BPO, 2% Gentamicin sulfate, 5% Vancomycin hydrochloride, colorant E141	98% Methyl methacrylate, 2% N,N-dimethyl-p-toluidine, hydroquinone, colorant E141

## Data Availability

Supplementary data can be obtained upon request from the authors.
